# Feasibility of a point-of-care test based on quantum dots with a mobile phone reader for detection of antibody responses

**DOI:** 10.1371/journal.pntd.0007746

**Published:** 2019-10-07

**Authors:** Chan Lee, John Noh, Seth E. O’Neal, Armando E. Gonzalez, Hector H. Garcia, the Cysticercosis Working Group in Peru, Sukwan Handali

**Affiliations:** 1 Medical College of Georgia, AU/UGA Medical Partnership, Athens, Georgia, United States of America; 2 Division of Parasitic Diseases and Malaria, Centers for Disease Control and Prevention, Atlanta, Georgia, United States of America; 3 School of Public Health, Oregon Health & Science University and Portland State University, Portland, Oregon, United States of America; 4 School of Veterinary Medicine, Universidad Nacional Mayor de San Marcos, Salamanca de Monterrico, Lima, Peru; 5 Department of Microbiology and Center for Global Health, Universidad Peruana Cayetano Heredia, Lima, Peru; 6 Cysticercosis Unit, Instituto Nacional de Ciencias Neurologicas, Lima, Peru; Consejo Nacional de Investigaciones Cientificas y Tecnicas, Fundación Mundo Sano, ARGENTINA

## Abstract

We developed a novel and portable fluorescent sensor that integrates a lateral flow assay with a quantum dot (Qdots) label and a mobile phone reader for detection of specific antibodies in human serum. We evaluated the utility of this assay to test for antibodies to the *Taenia solium* rT24H antigen. It was a retrospective study by examining 112 positive human sera from patients with neurocysticercosis (NCC) including samples from patients with single viable cyst (n = 18), two or more viable cysts (n = 71), and subarachnoid (racemose) cysts (n = 23). These samples were collected from previous study subjects in Lima, Peru under an approved study protocol in Peru. The sera were made anonymous under a protocol approved by the CDC Institutional Review Board. Definitive diagnosis of the subject was established by computed-tomography and/or magnetic resonance imaging. To test the specificity of the assay, we evaluated a panel of serum samples obtained from patients with other infections (n = 24), and serum samples from persons in the United States and Egypt who had not traveled outside their country, and therefore are presumed negative for cysticercosis (n = 128). The assay specificity in the negative panel was 99% (95–100%) while assay sensitivity was 89% (79–95%) in NCC patients with two or more viable cysts. Our assay has performance characteristics similar to those of traditional platforms for the detection of NCC and shows promise as a mobile phone reader-based point-of-care test for antibody detection.

## Author summary

Point-of-care (POC) assays are important tools in control and elimination of parasitic diseases such as lymphatic filariasis, malaria, and leishmaniasis. Most POC assays use immunochromatographic and lateral flow assay principles with gold nanoparticles as a reporter. Assays based on gold nanoparticles usually provide qualitative or semi-quantitative results and have relatively low sensitivity. However, other reporter alternatives are available, including quantum dots (Qdots), up-converting phosphor nanoparticles, and superparamagnetic particles. We developed a Qdots-based test in a lateral flow assay format with a mobile phone reader to detect antibody responses, using neurocysticercosis (NCC) as a disease model, and found that the performance is similar to the traditional platform for detecting antibody responses in subjects with NCC. The incorporation of the mobile phone reader offers the advantage of portability and adaptability for use in areas where laboratories are not immediately accessible. This novel POC assay with mobile phone reader is a feasible option for antibody responses detection.

## Introduction

Point-of-care (POC) assays, which can be performed at or near the site of care with a rapid turnaround time, are pivotal to transforming global disease control efforts, particularly in resource-constrained settings where access to laboratory facilities is limited. POC assays improve the management of patients by enabling immediate diagnosis and early treatment. [1, 2] Currently, most POC assays are based on immunochromatography in a lateral flow assay format using gold conjugate as a reporter that results in visible bands for a positive reaction. Visual reading of such bands is subjective and may led to false positive or false negative interpretation. [3] Furthermore, lateral flow assays are limited to qualitative or semi-quantitative results and often exhibit a relatively low sensitivity. [4]

Many alternative particles have been tested as reporters to improve the analytical performance of the traditional gold nanoparticle-based assays, including luminescent (e.g. Qdots, up-converting phosphor nanoparticles) and magnetic nanoparticles. [4–6] Qdots are a semiconducting, fluorescent nanoparticle with cadmium selenium core and zinc sulfide shell that can be coupled with proteins to be used as a fluorescent label. Qdots have advantageous properties including broad adsorption, narrow and symmetric photoluminescence spectra, high fluorescence intensity, and strong photo-stability. Qdots with wide ranges of wavelength emissions are available, including a maximum emission at 655 nm, a wavelength that is compatible with the use of a mobile phone reader. [4, 7]

Cysticercosis is an infection caused by the larval form of the pork tapeworm *Taenia solium*. When larval cysts form in the human brain (neurocysticercosis, NCC), they can result in seizures and other neurologic disorders.[8] In endemic areas, NCC is a frequent cause of late-onset seizures and epilepsy and ~30% of people with seizure disorders have neuroimaging findings suggestive of NCC. [9–12] NCC also affects those living in non-endemic countries, with an estimated more than 18,000 hospitalizations in the US between 2003 and 2012. [13]

In the absence of a pathologic specimen, definitive diagnosis of NCC requires neuroimaging. Serologic assays provide information that supports the overall diagnosis, particularly when images are not conclusive. Serologic assays may also play a role in endemic settings where access to neuroimaging is limited, by identifying patients for whom neuroimaging should be prioritized. The reference serological assay for the detection of NCC is the detection of *T*. *solium* antibodies using seven lentil lectin purified glycoprotein (LLGP) antigens in the enzyme-linked immunoelectrotransfer blot (EITB) format. This assay has very high sensitivity (>99%) and specificity (100%) in cases in which two or more viable cysts are present, but has lower sensitivity when there is a single viable cyst (reported sensitivity ranging from 52–79%). [6, 14, 15] One of the LLGP antigens, rT24H, performs very well in several formats, including Western blot, and multiplex-bead based assays. [5, 6, 15, 16] Existing platforms such as ELISA and Western blotting typically require hours to days to generate a result, which is impractical in endemic settings where patients often travel far to receive care, or where community screening is the only practical option. The well-documented performance of both the LLGP and rT24H antigens, combined with the knowledge of antibody responses in exposed and infected humans [17, 18] makes them particularly useful for developing novel POC methods. While two previous POC tests based on rT24H performed well, those tests rely on readers that are not readily available in resource poor settings. [5, 6]

Here we present a novel POC test using rT24h antigen, Qdots and a mobile phone reader, and evaluated the performance of this new assay using well-characterized sera from patients with NCC.

## Materials and methods

### Ethics statement

All clinical samples used in this study were collected in previous studies with specific permission for future use of stored samples (CDC Study Protocol Number 3580 and Universidad Peruana Cayetano Heredia, Lima, Peru Protocol Number 54702). Samples were anonymized and the study was performed in compliance with protocols approved by the ethical review boards of the CDC.

### Serum specimens

We used a panel of serum samples from 112 Peruvian patients with NCC to evaluate the sensitivity of the POC test. The diagnosis in all 112 cases was confirmed by either computed tomography and/or magnetic resonance imaging. The number, location, and stage of brain cysts was documented. Based on known differential antibody responses by type and burden of NCC, sera were separated into three mutually exclusive categories that have historically been used to evaluate serologic tests for NCC [6, 14–16]:

• Single viable cyst (n = 18)—samples from patients with only one viable brain cyst, regardless of whether additional degenerating or calcified cysts were present. A viable cyst was defined as a hypodense cystic lesion on CT or a cystic lesion with T2-hyperintense content, independent of the presence or degree of perilesional inflammation (thus including cysts with contrast enhancement and perilesional edema). [19] Sensitivity of antibody detection assays in individuals with a single brain cyst is usually low and ranges from 20 to 70% [6, 14–16]

• Two or more viable cysts (n = 71)—samples from patients who had two or more viable brain cyst, regardless of whether additional degenerating or calcified cysts were present. Sensitivity of antibody detection assays in individuals with two or more cysts is variable but higher than that in single cyst cases, expected to be between 80–99% [6, 14–16]

• Subarachnoid (racemose) cysts (n = 23)—samples from patients with subarachnoid /racemose cysts, regardless of whether additional viable, degenerating or calcified cysts were present. Subarachnoid / racemose NCC was defined by the presence of lesions in the basal subarachnoid spaces, the interhemispheric space, or the Sylvian fissure. Small cystic structures in the convexity of the cerebral hemispheres were not considered in this category. Antibody responses in subarachnoid NCC are usually very strong and consistently positive. [6, 14–16]

To evaluate the specificity of the POC test, we also assembled from our biobank a panel of serum samples (n = 152) from individuals presumed to be negative for NCC based on country of residence and travel history or lack of known previous diagnosis of NCC. These include sera obtained from healthy residents of countries not endemic for *T*. *solium* (The United States and Egypt) (n = 118 + 10 = 128). Serum donors from Egypt were tested by stool examination for intestinal parasites, and all were negative. Sera obtained from patients diagnosed with other parasitic infections in the CDC reference diagnostic (n = 24, consisting of *Schistosoma mansoni* [n = 5], 10 *S*. *haematobium* [n = 10], *Echinococcus granulosus* [n = 4], *E*. *multilocularis* [n = 1], *Endolimax nana* [n = 1], and *Wuchereria bancrofti* [n = 4],) were tested for cross-reactivity.

For optimization of the Qdots POC, a negative control serum pool was prepared by combining five serum samples from U.S. subjects not known to have NCC and who had not traveled outside the U.S. A positive control serum pool was similarly prepared by combining five serum samples from Peruvian patients with NCC.

### Qdots assay

#### Optimization of the assay

To develop the assay, we determined both the optimal concentration of T24H protein to couple to Qdot microspheres and the optimal amount of T24H protein sprayed onto the membrane test line. Different mole ratios (5–20 fold) of rT24H protein [4] were added to 8 μM of Qdots microspheres using the coupling protocol described below. Each of these couplings were tested against a strip containing 2 mg/mL T24H protein sprayed on the test line. The optimal concentration of T24H coupled to Qdots was determined based on the average three runs of the relative ratio of the results of the pooled positive sample to the results of the pooled negative sample.

Next, to determine the optimal concentration of T24H to dispense onto the test line, several concentrations of rT24H (0.5 mg/mL, 0.75 mg/mL, 1 mg/mL, and 2 mg/mL) in PBS were evaluated using the optimal coupling concentration as determined above. Determination of the optimal concentration of T24H coupled to Qdots was done by comparing the average three runs of the relative ratio of the results (through reading the fluorescence intensity of the line using the mobile phone reader) of the pooled positive sample against the pooled negative sample.

#### Preparation of rT24H-Qdot conjugate

To prevent precipitation during the pre-washing process, we activated 50 μL at a time of 8 μM Qdot 655 ITK carboxyl quantum dots (Cat. # Q21321MP, Molecular Probes Inc.; Eugene, OR) with 2-(N-morpholino)-ethanesulfonic acid (MES) buffered saline (50 mM MES, pH 5 + 0.85% sodium chloride [NaCl], 0.05% Tween-20). The Qdots were washed in Amicon Ultra 0.5 mL, 100 kDa molecular weight cut-off with MES-buffered saline at 8,500 g at 4 ^o^C for 3 minutes then suspended in 100 μL of MES buffered saline. Immediately, 11.5 μL of 10 mg/mL solution of 1-ethyl-3-[3-dimethylaminopropyl] carbodiimide hydrochloride (EDAC) (Cat. # 341006, Millipore, Billerica, MA) was added to the Qdots and mixed for 10 minutes at room temperature, followed by 11.5 μL of 10 mg/mL solution of Sulfo-NHS (Cat. # 24510, Thermo Scientific, Rockford, IL), which was also mixed for 10 minutes. The Qdots were then washed twice with MES buffered saline and another two times with only MES buffer.

For the coupling stage, the desired amount of rT24H protein [4] was added to 8 μM Qdot microspheres, and the total volume was brought up to 500 μL with MES buffer. This mixture was covered with aluminum foil to protect the photo-reactive nature of the Qdots and incubated for 2 hours at room temperature with end-over-end-mixing. Afterwards, the rT24H-Qdot complex was washed two times with MES buffered saline and two times in PBS-BN (1x PBS, pH 7.2 + 1% BSA, 0.05% sodium azide). The rT24H-Qdot complex was then brought up to a volume of 500 μL with PBS-BN and mixed gently for 30 minutes at room temperature with end-over-end mixing. Next, PBS-TBN (1x PBS, pH 7.2 + 1% BSA, 0.05% sodium azide + 0.05% Tween-20) + Pefabloc SC (0.1 mg/ml), pepstatin (0.7 μg/ml), and leupeptin (1 μg/ml) was used to complete a final wash and to dilute the rT24h-Qdot complex to an optimal concentration of 1.6 mg/mL.

#### Preparation of test strips

The test strips are composed of an absorbent cellulose fiber pad (Cat. # CFSP173000, Millipore, Billerica, MA), a nitrocellulose membrane (Cat. # MAMBR-0100, CNPC-SS12 15 U x 25 mm, Arista Biologicals, Allentown, PA), an Ahlstrom 8964 glass fiber conjugate pad (Cat. #MAPDS-0300, Arista Biologicals), a cellulose fiber sample pad (Cat. # CFSP001700, Millipore), and a plastic backing card (Cat. # MACRD-0201, backing card 60 x 300 mm, Arista). The sample pad was treated with a blocking buffer (0.1 M sodium tetraborate + 1% Triton X-100 + 1% polyvinylpyrrolidone (PVP), pH 9.3) briefly and air-dried in a fume hood overnight. The conjugate pad was treated with a separate solution of blocking buffer (0.5% polyvinyl alcohol (PVA) + 50 mM disodium phosphate, 0.1% Triton X-100 + 0.5% bovine serum albumin (BSA), pH 7.4) briefly and was also dried in a fume hood overnight.

#### Dispensing proteins to nitrocellulose membrane

The required concentrations of rT24H in PBS and Biotin-BSA (0.5 mg/mL) in PBS (Biotin-BSA, Cat. # AGBIO-0100, Arista Biologicals) proteins based on optimization results were sprayed onto the nitrocellulose membranes using IsoFlow Dispenser Model J008 (Imagene Technology, Lebanon, NH) at a volume of 0.1 μL/mm. The sprayed membranes were dried in an incubator at 37°C for one hour and stored at room temperature in a dry chamber (relative humidity <30%) until use.

#### Dispensing conjugates to treated conjugated pad

Qdot 655 Streptavidin Conjugate (Cat. # Q10121MP, Molecular Probes, Eugene, OR) at a concentration of 1 μM was mixed with the rT24H-Qdot conjugate at a ratio of 1:2. Immediately before spraying, 5% sucrose and 5% trehalose (w/v) were added to the conjugate mixture to improve flow when pushed through the lateral flow architecture with chase buffer. The IsoFlow Dispenser was used to dispense the Qdot conjugate mixture at 0.3 μL/mm at three psi onto the treated conjugate pad. The sprayed conjugated pad was dried in a fume hood overnight and stored in a dry chamber until use.

#### Assembly of the device

The absorbent pad, sprayed nitrocellulose membrane, sprayed conjugated pad, and treated sample pad were placed on a backing card with 1–2 mm overlap between each material to facilitate sample flow. A guillotine cutter (Index-Cutter II, A-Point Technologies, Gibbstown, NJ) was used to produce 3.5 mm strips, which were then placed in cassettes (Cat. # MACST-0100, 3.8 mm, Arista Biologicals).

#### Testing serum samples

For the test, 10 μL of a serum sample was pipetted onto the sample pad, quickly followed by 100 μL of PBS-Tween 0.3% as chase buffer. Within 30 minutes, the antibodies within the positive sample complex with the rT24h-Qdot conjugate and migrate through the nitrocellulose membrane, attaching to the test line where rT24H had been sprayed. The rT24h-Qdot complex is retained at the test line and the Streptavidin-Qdot conjugate migrated further to produce a control line where the biotin-BSA had been sprayed. In negative serum samples, with no antibody to complex with the Qdot conjugate, the rT24h-Qdot complex migrates past the test line on the nitrocellulose membrane, thereby producing only the control line with the Streptavidin-Qdot conjugate.

After 30 minutes, the lines were read using the Holomics Reader HRDR-30 vs 2.1.5. (Cellmic, Long Angeles, CA). The reader was calibrated manually using Test Developer Software version 1.0 (Cellmic, Long Angeles, CA) to allow the device to quantify the fluorescent signals at specific points along the test strip. Once the emitted wavelengths were quantified, the cellular device saved each strip's result so that it could be retrieved at a later point.

### Interpretation of results

To control for the variability in flow between cassettes, a ratio of the test line to the control line signals was calculated for each sample. Cut-off values that gave the optimal balance between sensitivity and specificity and assay performance characteristics (sensitivity and specificity) for each category of NCC were calculated with R statistical software version 3.4.1. (R Foundation for Statistical Computing, Vienna, Austria) and the pROC package. [16] Receiver operating characteristic (ROC) curves were created for each category of NCC-positive samples—all viable cysts, a single viable cyst, two or more viable cysts, and subarachnoid cysts. Samples with test line/control line values below the cutoff were classified as negative, while those samples with test line/control line values above the cutoff were classified as positive.

## Results

### Optimization of the POC test

**Fig 1:**
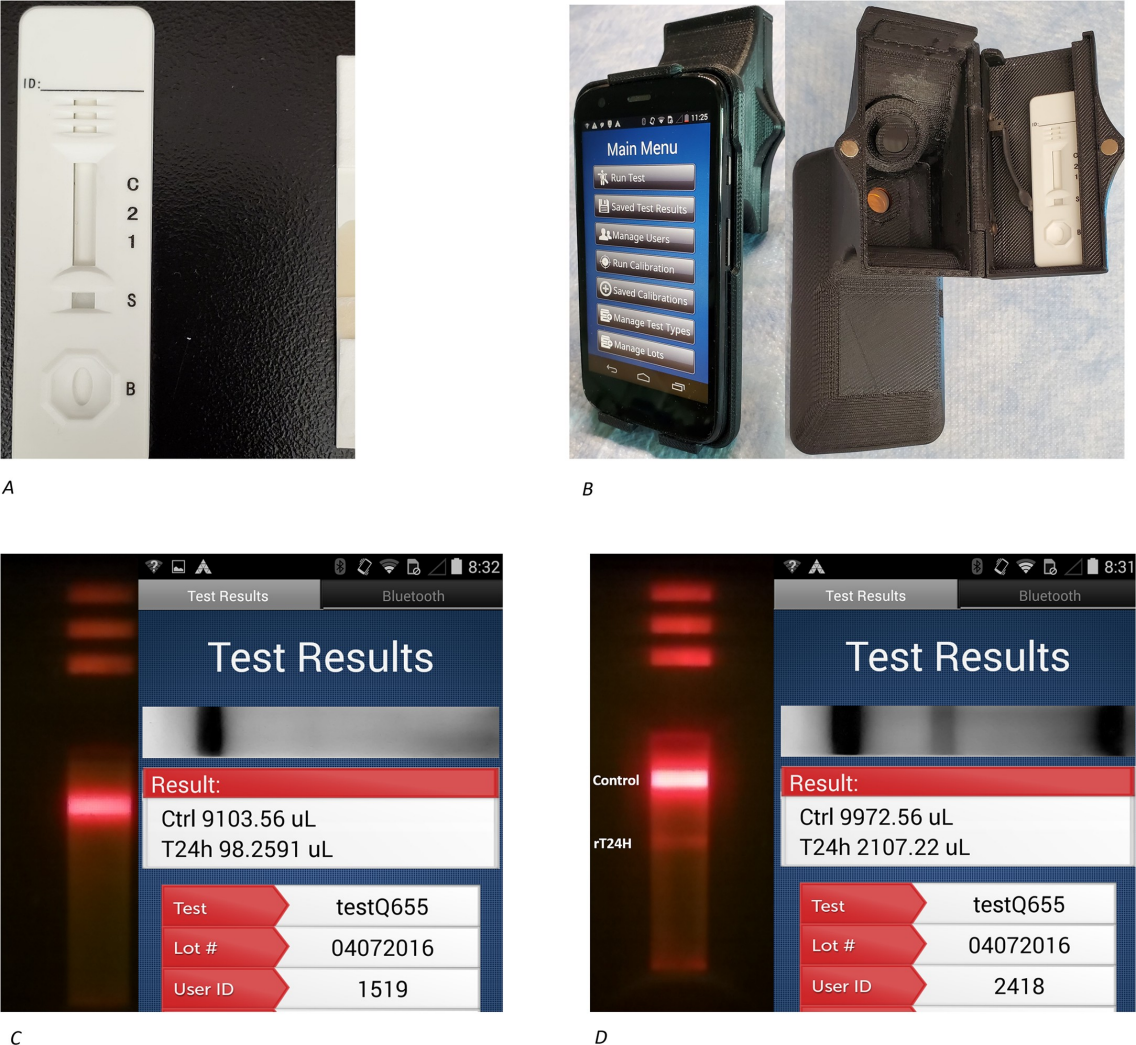
System view of the Qdot POC test device. A) Each 0.3 mm test strip was encased in one-time-use cassette and stored in a dry chamber to be read later with the mobile reader. B) The Holomics Reader HRDR-300 mobile device was used to measure fluorescent intensity of the Qdot complex after lateral migration of patient serum. C) Example of the result of a negative serum sample D) Example of the result of a positive serum sample.

Use of a 10-fold molar ratio–equivalent to 20 nmoles–of rT24H to Qdots resulted in the best signal-to-noise ratio. The amount of sprayed rT24H antigen that resulted in the best signal to noise ratio was 1 mg/mL of rT24H antigen. Fig 1 shows the optimized device, the reader, and illustrative negative and positive results.

### Performance (sensitivity and specificity) of the POC test

**Fig 2:**
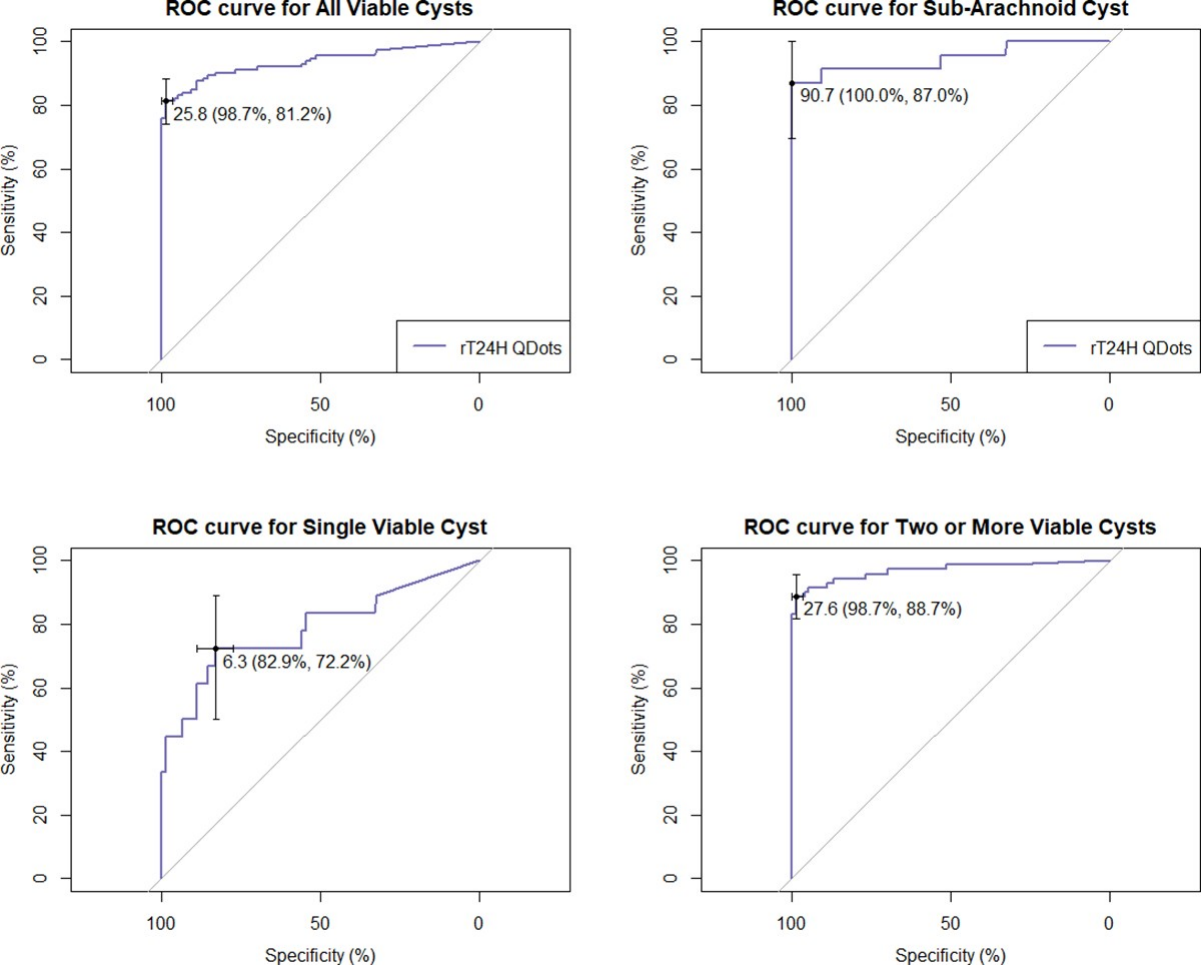
ROC curves for detection of total IgG responses in serum from patients with neurocysticercosis, using rT24H QDots based assay. Curves were developed using *pROC* package in R software version 3.3.3. 25.8 (98.7%, 81.2%) means at the cut-off point 25.8, the sensitivity is 81% and the specificity is 98.7%.

The performance of the POC test for sera from patients with two or more viable cysts and for subarachnoid/racemose cysts was better than that for samples of patients with single viable cyst (Fig 2). The cutoff value based on optimizing sensitivity and specificity of the ROC curve for the “two or more viable cysts” category was 27.6 (Fig 2). The sensitivity and overall specificity, all based on a cut-off point of 27.6, is shown in Table 1. The sensitivity was 89% when the analysis was limited to the group with two or more viable cysts and 87% when limited to the group with subarachnoid cysts. The sensitivity of the assay was lower (44%) for the category of single cyst. Overall combined sensitivity for the category of all viable cysts was 81%. The specificity of the assay for the negative panel was very high (99%), with cross-reactivity detected in only two samples (1 negative U.S. sample and a serum sample from a Nigerian patient with *Schistosoma mansoni*).

**Table 1:** Performance of rT24H POC test by category of NCC diagnosed using computed tomography (CT) or magnetic resonance imaging (MRI).

	Category			
	Single viable cyst	2 or more viable cysts	Subarachnoid cyst	All types of NCC
Sensitivity* (95% C.I.)	44 (22–69)	89 (79–95)	87 (20/23)	81 (73–88)
**Specificity** * (95% C.I.)	99 (95–100)			

* = based on 27.6 cut-off point (for 2 or more viable cysts)

## Discussion

Although there are several lateral flow assays (LFA), they share many of the same challenges and hurdles. Many of these tests require readings within a certain timeframe; the appearance of the test strips continues to change over time making it difficult or impossible to go back and read again. In most cases, at least two persons need to be trained to properly and consistently evaluate the tests. With the use of fluorescent Qdots and a mobile telephone reading device, an image is taken, and the test results are recorded with a hand-held device. This allows the review and evaluation of any test at a later time. The test can be performed by one person with basic understanding in loading samples into the cassette and an application on a smart-phone to operate and to take readings of the fluorescent Qdots POC test. We developed a POC test using fluorescent Qdots and a mobile telephone reading device that performs similarly those using other platforms for serological diagnosis of NCC (Table 2). Our assay has good sensitivity for two or more viable and racemose cysts but not for detection of a single viable cyst. The test was developed specifically for use in regions where sophisticated laboratory infrastructure and expertise may not be available but where POC testing is needed to inform appropriate clinical management or referral. The use of fluorescent microspheres allows the use of a cell phone-based reader with a lightweight 3D printed chamber that attaches to a standard cellular device. Advantages of a POC cellular device include ability to 1) make a timely diagnosis allowing rapid management decisions while the patient is still present, 2) conduct the assay without the need of a centralized lab or highly trained personnel, 3) obtain quantitative data which have the potential to be used to triage severity of infection quickly and efficiently, and 4) allow such data to enter a cloud for immediate collection and study of disease management and prevalence in endemic areas.

**Table 2:** Comparison of the performance of rT24H POC against other assay platforms.

	Qdots	Up-converting Phosphor Particle [5]	Magnetic Immuno-chromatographic Test [6]	Quick-ELISA [14]	Multiplex Bead Assay [16]	ELISA [16]	rT24H immuno-blot [15]	LLGP Immuno- Blot [15]
**Sensitivity**								
Single viable cyst	44		33	47–67			44–64	52–79
Two or more viable cysts	89	89–94	93	96–95	96	88	96–99	96–100
**Specificity**	99	98–100	99	95–96	97	97	97	97

The performance of the rT24H Qdot POC compared to other serological tests is shown in Table 2. All tests except the reference diagnostic LLGP assay use rT24H as the antigen. Performance of the rT24H Qdot POC equaled or approached that of other serologic assays testing for antibody to rT24H. The tests with the highest sensitivity–the multiplex bead assay, immunoblot, and the LLGP reference test–are also the most cumbersome or technologically advanced tests that are more challenging to use in remote or poor settings. A point of care screening test would be ideal in these settings to screen and prioritize individuals for neuroimaging. The rT24H POC also compares favorably to other POC tests, such as the magnetic immunochromatographic test (MICT) or the up-converting phosphor (UCP) test. The MICT had 33% sensitivity to detect a single viable cyst samples and 93% sensitivity, with 99% specificity, for persons with two or more cysts. [5] The MICT requires a liquid conjugate, thus requiring a higher number of steps to perform which makes this assay potentially more liable to human error in the field. In the Qdot POC assay described here, drying the conjugate onto the pad not only simplified the diagnostic process but also eliminated the need for a cold chain. The MICT is dependent on a benchtop reader for quantitative results, diminishing the accessibility of such a test; that challenge is overcome for the Qdot POC assay by using the mobile phone reader. The UCP test had 96% sensitivity and 97% specificity in cases with two or more cysts.

A screening test for NCC would ideally have very high sensitivity, so this test may need further optimization to be ideal for this usage. rT24H is an immunodominant antigen identified from LLGP bands, but use of a single antigen may not be sufficient to obtain the sensitivity desired for a POC screening test for individuals needing neuroimaging for NCC. Next steps will be to evaluate combinations of antigens that may increase the sensitivity of this test.

Our novel POC test based on a combination of rT24H antigen and Qdots for antibody detection has similar performance to that of other assays for NCC, but requires further optimization for use as a program tool for screening for NCC. Our experience demonstrates the feasibility for this test format and demonstrates the important potential use of mobile readers for qualitative reading of POC tests. It opens the way for point of care diagnosis in cysticercosis control program. Anti-parasitic therapy and other specific measures would require referral for neuroimaging not only as a confirmatory diagnostic tool but also to provide information on the location, burden and other characteristics of the parasitic lesions in the CNS as stated in the recent IDSA/ASTMH guidelines. [20, 21] Once test performance is well established, this test would add into the category of major clinical/exposure criteria ("*Detection of specific anticysticercal antibodies or cysticercal antigens by well-standardized immunodiagnostic tests*") in the recognized Del Brutto diagnostic criteria chart. [20, 22, 23]

This format, with generation of a rapid result through use of a cell phone reader-based assay, could be useful for field-based diagnosis, clinical management and control of neurocysticercosis or other neglected tropical diseases.

## Disclaimer

Use of trade names is for identification only and does not imply endorsement by the Public Health Service or by the US Department of Health and Human Services. The findings and conclusions in this report are those of the authors and do not necessarily represent the official position of the Centers for Disease Control and Prevention.

Members of the Cysticercosis Working Group in Peru include: Robert H. Gilman, MD, DTMH; Armando E. Gonzalez, DVM, PhD; Hector H. Garcia, MD, PhD; Victor C.W.Tsang, PhD; Manuela Verastegui, PhD; Mirko Zimic, PhD; Javier A. Bustos, MD, PhD; Seth E. O’Neal, MD, MPH (Coordination Board); Silvia Rodriguez, MSc; Isidro Gonzales, MD; Herbert Saavedra, MD; Sofia Sanchez, MD; Manuel Martinez, MD (Instituto Nacional de Ciencias Neurológicas, Lima, Perú); Saul Santivanez, MD, PhD; Holger Mayta, PhD; Yesenia Castillo, MSc; Monica Pajuelo, PhD; Gianfranco Arroyo, DVM (Universidad Peruana Cayetano Heredia, Lima, Perú); Maria T. Lopez, DVM, PhD; Luis Gomez, DVM; Ana Vargas, DVM; Cesar M. Gavidia, DVM, PhD (School of Veterinary Medicine, Universidad Nacional Mayor de San Marcos, Lima, Perú); Luz M. Moyano, MD; Ricardo Gamboa, MSc; Claudio Muro; Percy Vichez, MSc (Cysticercosis Elimination Program, Tumbes, Perú); Sukwan Handali, MD; John Noh (Centers for Disease Control, Atlanta, USA); Theodore E. Nash MD (NIAID, NIH, Bethesda, MD); Jon Friedland (Imperial College, London, UK).

## Supporting information

S1 Checklist (DOCX)

S1 File Testing Defined Sera 28MARCH2019.xlsx file contain the list of all the defined sera used for assay performance determination. In the disease category column, description and number of cysts are presented.
(XLSX)
